# The 1, 2-ethylenediamine SQ109 protects against tuberculosis by promoting M1 macrophage polarization through the p38 MAPK pathway

**DOI:** 10.1038/s42003-022-03693-2

**Published:** 2022-07-28

**Authors:** Mona Singh, Santosh Kumar, Baldeep Singh, Preeti Jain, Anjna Kumari, Isha Pahuja, Shivam Chaturvedi, Durbaka Vijay Raghava Prasad, Ved Prakash Dwivedi, Gobardhan Das

**Affiliations:** 1 Special Centre for Molecular Medicine, Jawaharlal Nehru University, New Delhi, India; 2 Deshbandhu College, University of Delhi, Kalkaji, New Delhi, 110 019 India; 3 Immunobiology Group, International Centre for Genetic Engineering and Biotechnology, New Delhi, India; 4 Department of Microbiology, Yogi Vemana University, Kadapa, India

**Keywords:** Tuberculosis, Bacterial pathogenesis

## Abstract

Directly Observed Treatment Short-course (DOTs), is an effective and widely recommended treatment for tuberculosis (TB). The antibiotics used in DOTs, are immunotoxic and impair effector T cells, increasing the risk of re-infections and reactivation. Multiple reports suggest that addition of immune-modulators along with antibiotics improves the effectiveness of TB treatment. Therefore, drugs with both antimicrobial and immunomodulatory properties are desirable. *N*^1^-(Adamantan-2-yl)-*N*^2^-[(2*E*)-3,7-dimethylocta-2,6-dien-1-yl]ethane-1,2-diamine (SQ109) is an asymmetric diamine derivative of adamantane, that targets Mycobacterial membrane protein Large 3 (MmpL3). SQ109 dissipates the transmembrane electrochemical proton-gradient necessary for cell-wall biosynthesis and bacterial activity. Here, we examined the effects of SQ109 on host-immune responses using a murine TB model. Our results suggest the pro-inflammatory nature of SQ109, which instigates M1-macrophage polarization and induces protective pro-inflammatory cytokines through the p38-MAPK pathway. SQ109 also promotes Th1 and Th17-immune responses that inhibit the bacillary burden in a murine model of TB. These findings put forth SQ109 as a potential-adjunct to TB antibiotic therapy.

## Abstract (web summary)

The adamantine derivative SQ109 induces protective pro-inflammatory cytokines and promotes Th1 and Th17-immune responses that inhibit bacterial burden in a tuberculosis mouse model.

## Introduction

*Mycobacterium tuberculosis (M.tb)*, an intracellular pathogen that causes tuberculosis (TB), has co-evolved with its human host to facilitate its survival and transmission. TB remains a leading cause of death worldwide, with 10 million infected individuals and ~2 million new cases and 1.45 million deaths annually^1^. Nearly all countries, irrespective of their socio-economic status, are under threat from drug-resistant TB. Global Tuberculosis Report 2020, indicated that approximately 0.5 million people developed rifampicin-resistant TB (RR-TB) in 2019, of which 78% were multidrug-resistant TB (MDR-TB) (https://apps.who.int/iris/bitstream/handle/10665/336069/9789240013131-eng.pdf). At this rate of conversion, all newly reported cases may convert into MDR by 2032, unless a major intervention is implemented. This situation is even more concerning when the pathogen acquires further resistance to become extensively drug-resistant (XDR). It is estimated that 4.1 percent of current MDR cases are XDR TB^1^. Recently, a totally drug-resistant (TDR) strain of *M.tb*, also known as extremely-drug-resistant (XXDR) TB, which exhibits resistance against all known antibiotics, has emerged^2,3^. Therefore, novel strategies should be devised to treat patients infected with these resistant *M.tb* strains.

The current TB treatment regimen, Directly Observed Treatment Short-course (DOTs), is lengthy and causes various side effects like hepatotoxicity, dyspepsia, arthralgia, and exanthema^4,5^. Additionally, the frontline antibiotics like isoniazid in DOTs therapy exhibit immunotoxic properties, which eliminate antigen-responding T-cells and make patients vulnerable to re-infection and disease re-activation^4^. Therefore, new therapeutic regimens that reduce the length of treatment, less toxic, and exhibit immuno-protection are highly desirable. These therapeutic regimens might be useful to support WHO -END TB strategy.

In recent years, the 1, 2-ethylenediamine (SQ109) has emerged as a new and promising TB drug. It was developed through a combinatorial chemistry program upon screening of 63,238 drug candidates. This drug discovery strategy was designed around the 1, 2-ethylenediamine pharmacophore of Ethambutol^6^. SQ109 exhibits satisfactory antibacterial activity against both drug-sensitive and -resistant *M.tb* strains^7^. The known mode of action of both SQ109 and isoniazid (INH) is to interfere with the assembly of mycolic acids, a component of cell wall core of *Mycobacterium tuberculosis*^8,9^. To accomplish this, SQ109 targets Mmpl3 which is a transporter of mycobacterial trehalose monomycolate (TMM), a precursor of trehalose dimycolate (TDM) and cell wall mycolates^9^. In contrast to this, INH mode of action involves enzymatic oxidation of INH by the catalase-peroxidase enzyme (KatG) to form isonicotinoyl radicals, which leads to the formation of a key chemical adduct with nicotinamide adenine dinucleotide (INH-NAD^+^)^10^. This adduct then inhibits enoyl acyl-carrier-protein reductase, an essential enzymatic component of mycolic acid biosynthesis^10^. In addition to this, ethambutol, which is the parent molecule of SQ109, also affects the synthesis of mycobacterial cell wall via targeting the membrane-associated arabinosyl transferases, EmbA and EmbB and inhibiting the polymerization of arabinogalactan^11^.

Furthermore, SQ109 has synergistic activities with other conventional anti-tubercular drugs such as INH, rifampicin and bedaquillin, and thereby reduces the length of treatment by 30% or more^12–14^. In addition, a recent study indicated that a new regimen constituting SQ109 achieved a relapse free cure in 3 weeks in murine model of tuberculosis^15^. SQ109 has successfully completed phase 1 and phase 2 clinical trials for treatment of TB^13,16–18^, and the Food and Drug Administration (FDA) approved its fast track designation and orphan drug status. Based on these clinical studies SQ109 is a highly promising adjunct drug for TB treatment.

Immunopathogenesis and generation of MDR/XDR strains of TB necessitate the development of adjunct immunotherapy for the treatment of resistant and sensitive TB. Studies have shown that adjunctive immunotherapy along with conventional therapy is effective in shortening TB treatment and related pathologies. Previously we have shown that immunomodulators such as curcumin nanoparticles, Clofazamine, and Luteolin help the restoration of antigen-specific T cell proliferation along with reduced apoptosis in T cells^19–22^. Hence, addition of immunomodulators reduces the possibility of reinfection and reactivation^19^ of the disease. Similarly, phytochemical bergenin, an active component of leaves of Shorea robusta, induces Th1 and Th17 cells based protective immune responses^23^. Along with this, bergenin has been shown to potentially inhibit the mycobacterial growth in a murine model of *M. tb* infection^23^.

Additionally, studies have shown that the antibiotics such as Bedaquiline can interfere with the host immune system, indirectly through the dysbiosis of gut microbiota^24^, or directly by modulating the functions of immune cells^25^. Therefore, we examined the immunomodulatory effects of SQ109, a promising anti-mycobacterial drug by studying the dynamics of cytokines and effector immune cells in a murine model of TB. We demonstrated the protective pro-inflammatory nature of SQ109 by studying the polarization status on macrophages upon SQ109 treatment. Additionally, our findings revealed that SQ109 treatment induces apoptosis in *M.tb* infected macrophages. Taken together, this study explores how SQ109 modulates host immune response using both in vitro and in vivo models of TB infection.

## Results

### SQ109 treatment reduces the bacterial load in mice infected with *M. tuberculosis*

**Fig. 1:**
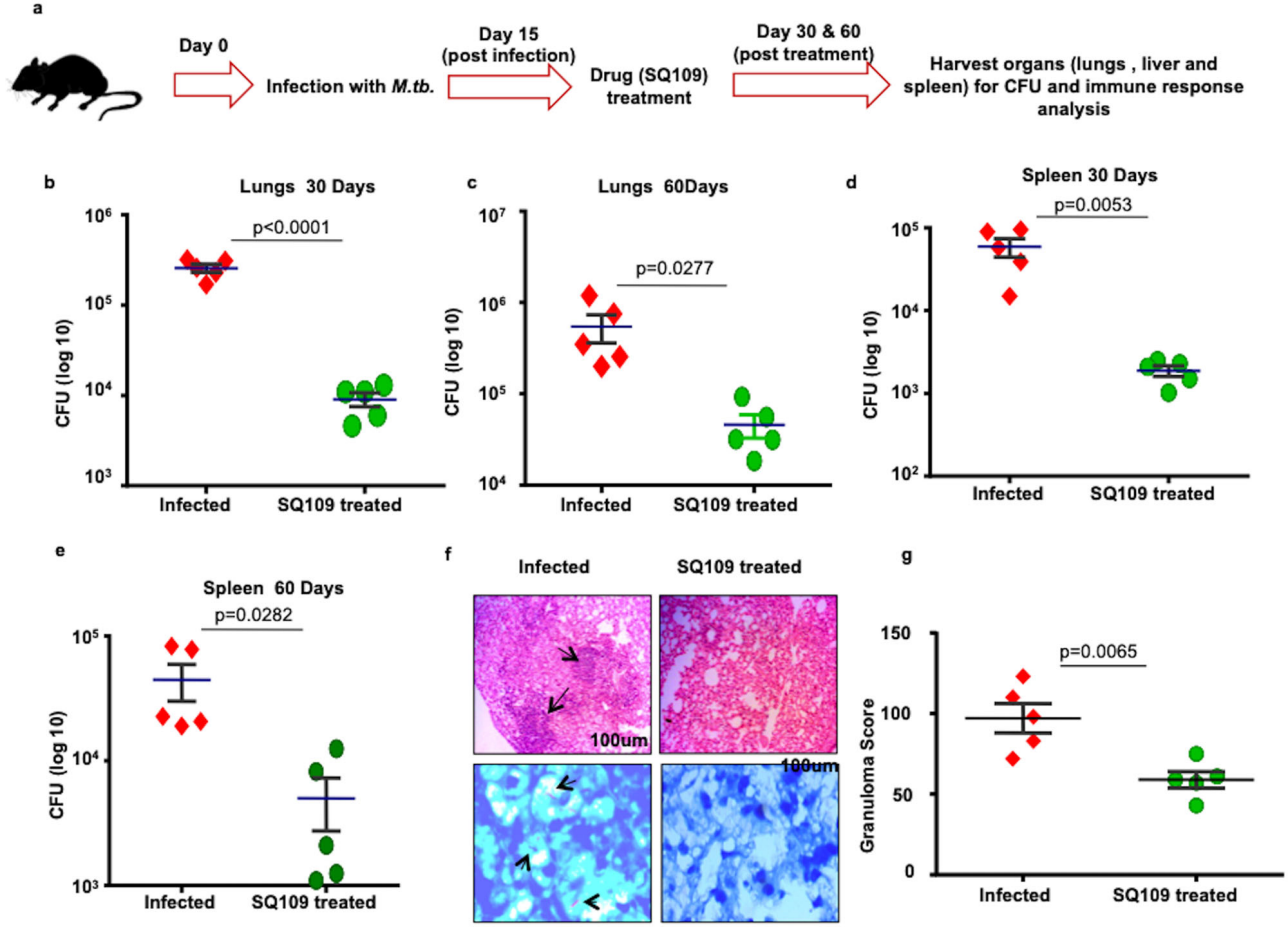
SQ109 treatment reduces the bacterial burden in a murine model of tuberculosis. **a** Schematic diagram to show different groups of C57BL/6 mice challenged with H37Rv strains of *M.tb* via the aerosol route with a low-dose inoculum of ~110 CFU/mouse. After 15 days groups of mice were treated daily with SQ109 (10 mg/kg) for 30 or 60 days. Mice were sacrificed and lungs and spleen were harvested for estimation of bacterial burden and analysis of immune responses. **b**, **c** CFU from lung homogenates at 30 and 60 days post-treatment. **d**, **e** CFU from spleen homogenates at 30 and 60 days post-treatment. **f** Lungs from the different groups of mice were harvested, preserved in 4% paraformaldehyde, sectioned and stained with Hematoxylin and Eosin (H&E) and AFB. (Arrows indicate granulomatic lesions and bacilli in infected mice). **g** Bar diagram to show the number of granulomas in infected and treated mice. *n* = 5 biological replicates were used for two independent experiments. Statistical significance was determined using unpaired *t*-test.

Previous studies have shown that SQ109 significantly reduces bacterial loads in lungs and spleen over a treatment time of 28 days in a murine model of TB^7^. However, the effects of SQ109 treatment on host immune responses during *M.tb* infection remain incompletely understood. To explore this aspect, we infected C57BL/6 mice with a low dose of *M.tb* H37Rv (~110 CFU) via the aerosol route and treated the animals daily with SQ109 (10 mg/kg body weight) starting from the 15th day of post infection until sacrifice^12,21,23^ (Fig. 1a). At various time points of post treatment, we harvested lungs and spleen to determine bacterial burdens and immune responses. We found that SQ109 treatment significantly reduces the bacterial burden by ~ 1.5 logs (Fig. 1b–e), which is in agreement with previous reports. These observations were further supported by histological analysis of lungs, which revealed that a reduced numbers of inflammatory granulomatous and necrotic lesions in SQ109-treated mice (Fig. 1f, g). These data confirm the anti-microbial activity of SQ109.

### SQ109 augments protective immunity against *M.tb*

**Fig. 2:**
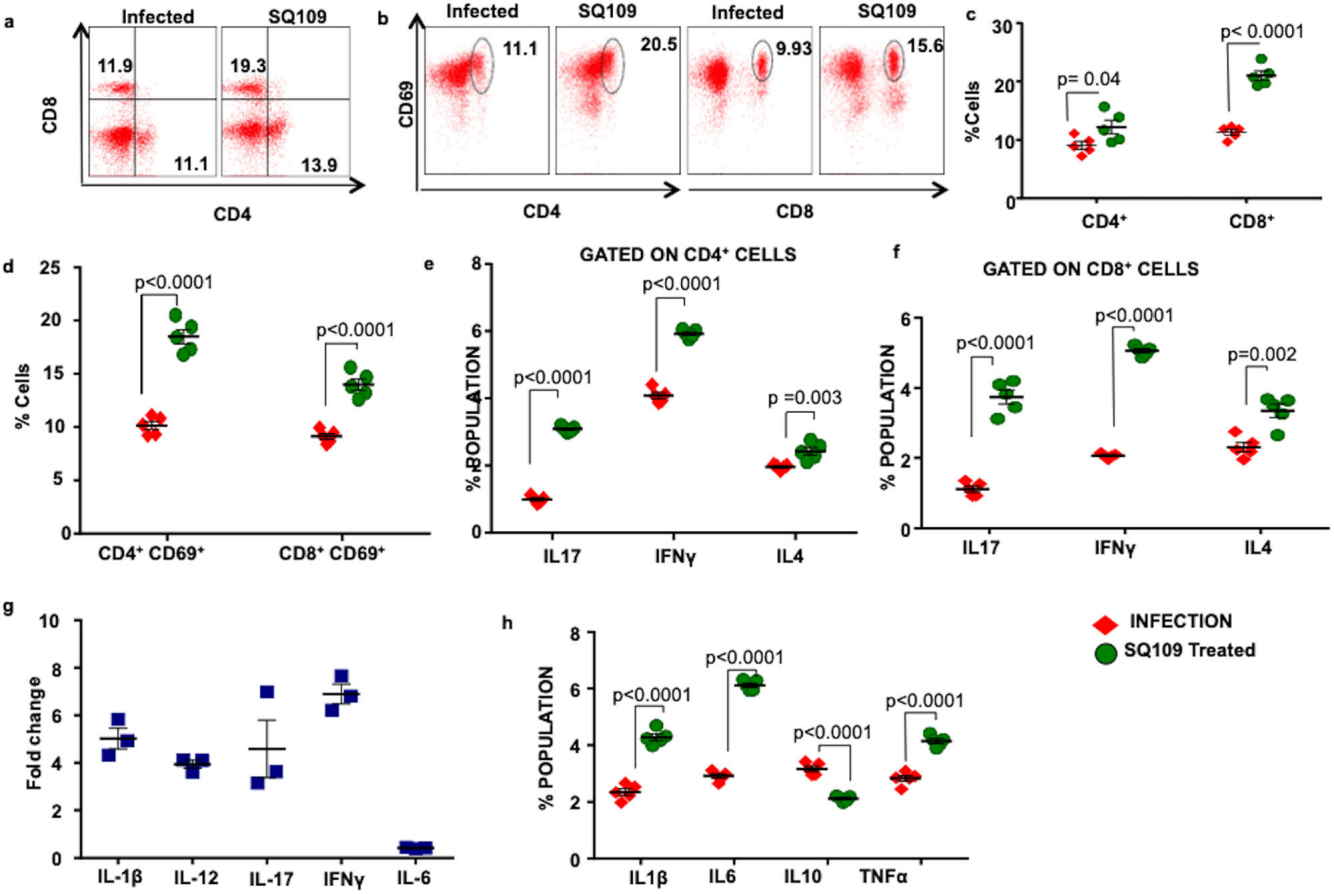
SQ109 treatment induces protective immunity against *M.tb*. **a**, **c** FACS data to show the percentage of CD4^+^ and CD8^+^ T cells in the spleen of different groups of mice infected with the H37Rv strain of *M.tb* and treated with SQ109. **b**, **d** Activation profile of T cells (CD4^+^ and CD8^+^) in spleen of mice infected with H37Rv and treated with SQ109. **e** Cytokine profile (IFN-γ, IL-17, and IL-4) of CD4^+^ T cells in the spleen of different groups of mice infected with H37Rv and treated with SQ109. **f** Cytokine profile (IFN-γ, IL-17 and IL-4) of CD8^+^ T cells in the spleen of different groups of mice infected with H37Rv and treated with SQ109. **g** RT-PCR data to show expression of cytokines at the transcriptional level in the lungs of different groups of mice infected with H37Rv and treated with SQ109. **h** Profiling of intracellular cytokines (IL-1β, IL-6, IL-10 and TNF-α) in splenocytes of different groups of mice infected with H37Rv and treated with SQ109. Data shown here are representative of two independent experiments with 5 mice in each group. Statistical significance was determined using multiple *t*-test. *n* = 5 biological replicates were used for two independent experiments.

The host immune system plays a vital role in containment of *M.tb* and only a fraction of infected individuals (approximately 10%) develop active TB^26^. It is well documented that IFN-γ-producing CD4^+^ T cells mediate protective immunity against TB^27^ and any defect in Th1 cytokine signaling (e.g., IFN-γ or IL-12 signaling) is reflected early during *M.tb* infection by a high bacterial load^27–29^. Inhibition of Th1 cell differentiation by blocking IL-12 production in infected macrophages is a well-known immune escape mechanism of *M.tb*^30–32^. The role of Th17 cells in host-protective immune responses against *M.tb* has recently been established^33^. Consequently, vaccine strains that are unable to mount Th17 responses lack significant vaccine efficacy^33^. In sharp contrast, IL-4-producing Th2 cells and regulatory T (Treg) cells may assist in the progression of *M.tb* infection by inhibiting protective Th1 and Th17 responses^34–38^. Hence, a dynamic balance between the anti-microbial and immunomodulatory activities of candidate drugs is preferred to eliminate *M.tb* infection and avoid the emergence of drug-resistance. Therefore, we examined whether SQ109 modulates host immune responses in *M.tb* infection. We observed a significant increase in CD4^+^ and CD8^+^ T cells in the spleen of SQ109-treated mice (Fig. 2a, c). These data suggest that SQ109 treatment reduces the immune dampening effects of antibiotics used in TB treatment^4^. We further determined the activation status of T cells, (Supplementary Fig. 1a, b), and found that SQ109 treatment leads to the activation of both CD4^+^ and CD8^+^ T cells, as revealed by increased CD69 surface expression (Fig. 2b, d). Moreover, we also determined the cytokines produced by T cells and the innate cytokines produced by phagocytes such as macrophages that are known to influence T cell differentiation during *M.tb* infection. We found that SQ109 treatment enhances the capacity of splenic CD4^+^ and CD8^+^ T cells from *M.tb*-infected mice to produce IFN-γ and IL-17 (Fig. 2e, f), which are considered major players in providing protective immunity against TB^26,27,33^. However, we found only modest effects on IL-4-producing CD4^+^ and CD8^+^ T cells (Fig. 2e, f). These findings clearly suggested that SQ109 enhances protective immunity by up-regulating Th1 and Th17 responses.

Next, we explored the effect of SQ109 on the dynamics of cytokine responses at both the transcriptional and translational level. Surprisingly, we found that SQ109 treatment potently induces the proinflammatory cytokines IL-1β, IL-6, and TNF-α, while down-regulating the anti-inflammatory cytokine IL-10 (Fig. 2g, h). It has been well documented that mice deficient in IL-1, IL-6, and TNF-α succumb early during *M.tb* infection and, hence, these cytokines are associated with protective inflammatory responses during infection^39–41^. Collectively, our data indicated that SQ109 enhances protection against *M.tb* infection by modulating the dynamics of protective host immune responses.

### SQ109 polarizes macrophages towards M1 phenotype

**Fig. 3:**
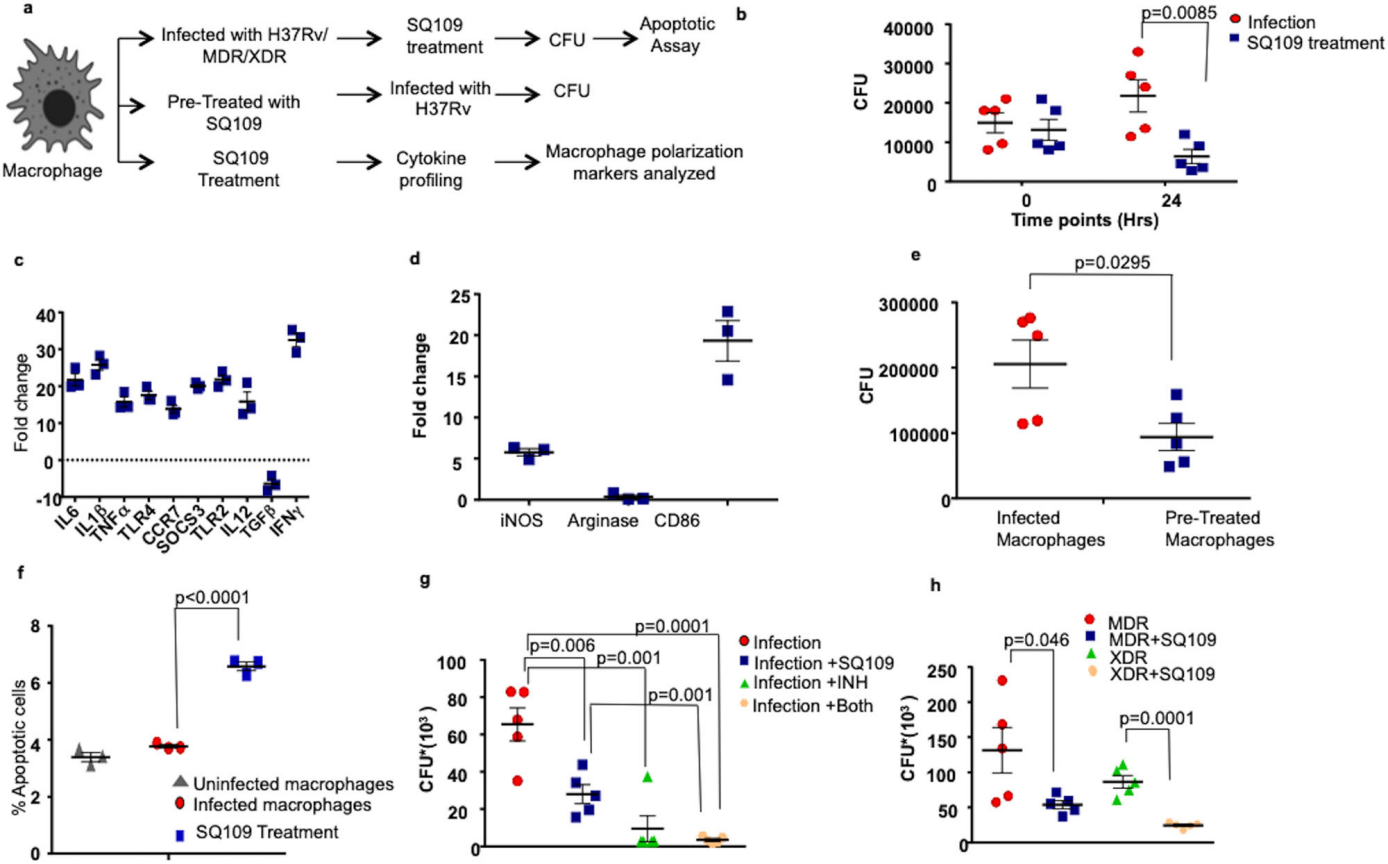
SQ109 treatment polarizes macrophages towards a protective M1-type. **a** Experimental layout: (i) Peritoneal macrophages were infected with different strains of *M.tb* followed by SQ109 treatment. After 24 and 48 h of treatment, cells were harvested for CFU determination and apoptotic assay. (ii) Macrophages were pre-treated with SQ109 (0.39 μg/ml) for 24 h, then washed, followed by H37Rv infection. At 24 h after infection, cells were harvested for CFU determination. (iii) Macrophages were treated with SQ109 followed by profiling of cytokines and macrophage-polarizing markers at 12 h and 24 h of treatment. **b** CFU data showing bacterial survival in macrophages infected with H37Rv and treated with SQ109 (0.39 ug/ml). **c**, **d** RT-PCR data showing fold-changes in the expression of cytokines and macrophage polarizing markers in SQ109-treated macrophages compared to control macrophages. **e** CFU data showing bacterial survival in macrophages pre-treated with SQ109. **f** Annexin V and Propidium Iodide FACS analysis of macrophages infected with H37Rv followed by SQ109 treatment. **g** CFU data showing bacterial survival in macrophages infected with H37Rv and treated with SQ109 (0.39 μg/ml) and Isoniazid. **h** CFU data showing bacterial load in macrophages infected with MDR & XDR strains of *M.tb* and treated with SQ109 (0.78 μg/ml). *n* = 3 biological replicates were used for three independent experiments. Statistical significance was tested using multiple/unpaired *t*-test unless specified otherwise.

Although multiple inflammatory mediators influence the progression of *M.tb* infection, macrophages and dendritic cells are the key cellular sources of cytokines that impact the initiation of both innate and adaptive immunity^42^. Because our study indicated that SQ109 promotes proinflammatory cytokine production in vivo, we next tested its effect on macrophages ex vivo. We infected peritoneal macrophages with virulent strain of *M.tb* H37Rv, followed by SQ109 treatment, and then calculated the CFUs after 24 h (Fig. 3a). We observed that SQ109 significantly reduces the bacterial burden in macrophages (Fig. 3b).

Previous studies have shown that M1 macrophages promote acute inflammatory responses by producing proinflammatory cytokines such as IL-1β, IL-6, IL-12, and TNF-α, which mediate resistance against *M.tb*^43,44^. Therefore, we examined the effect of SQ109 on the polarization status of macrophages (Fig. 3a). We treated macrophages with SQ109 and examined cytokine production at 12, 24, and 48 h. We noticed significant transcriptional up-regulation of the M1-specific markers IL-6, IL-1β, TNF-α, IL-12, TLR-2, TLR-4, CCR7, SOSC3, IFN-γ, i-NOS, and CD86, concomitant with down-regulation of the M2-specific markers TGF-β and arginase (Fig. 3c, d and Supplementary Fig. 2). These data suggested that SQ109 polarizes macrophages towards a protective M1 phenotype, which promotes *M.tb* clearance.

To confirm the impact of SQ109 on *M.tb* clearance by macrophages, we pre-treated the macrophages for 24 h with SQ109 followed by infection with H37Rv (Fig. 3a). We registered that SQ109 pre-treated macrophages exhibited significantly reduced bacterial burdens (Fig. 3e).

Recent studies have indicated that the M2 phenotype predominates in macrophages infected with the virulent H37Rv strain of *M.tb*, as their mannose and scavenger receptors facilitate entry and M2-mediated IL-10 secretion, which inhibit macrophage apoptosis^45^. Therefore, we also assessed the effect of SQ109 treatment on macrophage apoptosis (Fig. 3a). We infected macrophages with H37Rv followed by SQ109 treatment and determined apoptosis levels at 48 h by annexin V and propidium iodide staining. Interestingly, we found that SQ109 augmented apoptosis in H37Rv-infected macrophages as compared with uninfected control macrophages (Fig. 3f). Taken together, these data indicated that SQ109 augments clearance of *M.tb* by inducing a macrophage-mediated protective environment.

We further studied the effect of SQ109 on H37 Rv, MDR, and XDR strains of *M.tb* in infected macrophages. We found a significant reduction in bacterial load in SQ109-treated macrophages post 48 h of infection (Fig. 3g, h). Therefore, SQ109 is a candidate drug for treatment of drug-resistant TB. Finally, we explored the effect of SQ109 at 48 h in combination with conventional anti-tubercular therapy in HR37Rv-infected macrophages. The results were in concordance with previous studies demonstrated by Nikonenko et al. 2007, as SQ109 exhibited synergistic effects with anti-tubercular drugs to significantly reduce bacterial load^12^ (Fig. 3g).

### SQ109 mediates pro-inflammatory responses via the p38 MAPK pathway

**Fig. 4:**
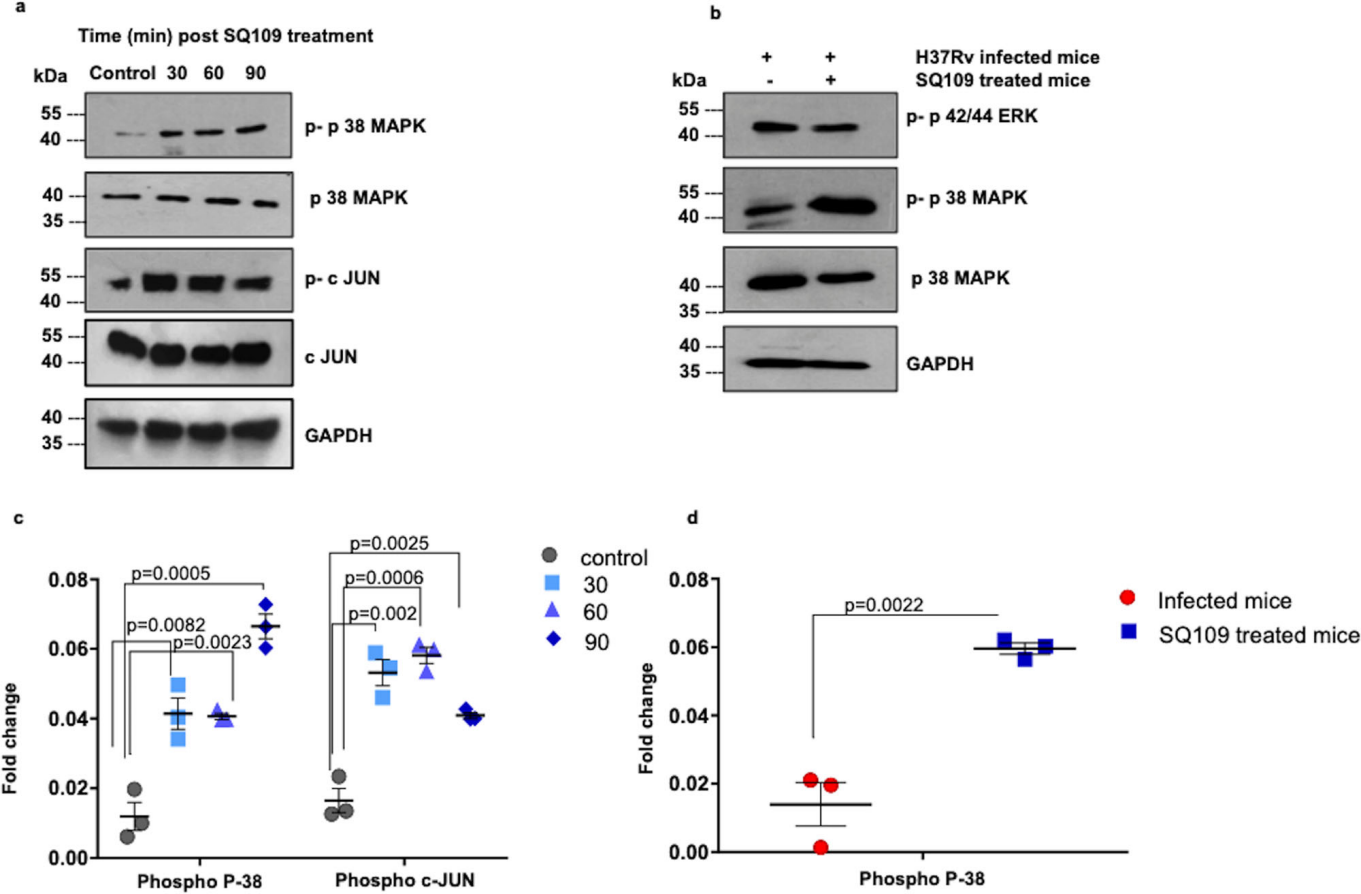
SQ109 treatment activates the MAPK pathway. **a** Phospho p38 and phospho-c-JUN protein levels were determined in peritoneal macrophages treated with SQ109 (0.39 μg/ml) for varying time periods (30, 60, and 90 min). Macrophages without SQ109 treatment were used as control. GAPDH was used as a loading control. **b** Phosphorylation of p38 MAPK and ERK1/2, evaluated by Western blotting in the cells isolated from lungs of different groups of mice infected with H37Rv and treated with SQ109 (10 mg/kg body weight). GAPDH was used as a loading control. **c** Summary of the phospho-p38 MAPK and phospho-c-Jun data from A. **d** Summary of the phospho-p38 MAPK data from B. *n* = 3 biological replicates were used for three independent experiments except for Fig. 4b, where only two independent experiments were undertaken. Statistical significance was tested using unpaired *t*-test.

To gain insight into the molecular mechanisms of SQ109-mediated immune modulation, we explored MAPK and JNK signaling pathways in macrophages, as they play critical roles in the production of pro-inflammatory cytokines that modulate macrophage polarization^1,46–50^. Furthermore, MAPK phosphorylation is related to inhibition of *M.tb* growth in macrophages^51^. MAPKs are also associated with Th1 cell activation and differentiation, which is critical for protection against *M.tb*. Anti-tubercular antibiotics, like isoniazid, is also known for activating p38 MAPK protein in time and dose dependent manner, while ethambutol which is a parent molecule of SQ109 is known to function via PKC signaling pathway rather MAPK pathway^52,53^. To elucidate the effect of SQ109 on MAPK and JNK pathways, we treated macrophages with SQ109 for various time points (30, 60, and 90 min) and assessed the expression of MAPK and JNK pathway proteins (Fig. 4a, c and Supplementary Fig. 3). Interestingly, we also found that SQ109 induces the phosphorylation of p38 MAPK and c-JUN (Fig. 4a, c).

Furthermore, to confirm SQ109-directed p38 MAPK activation in vivo, we isolated lung tissues from SQ109- and sham-treated, H37Rv-infected mice and analyzed the phosphorylation status of p38 MAPK protein. Interestingly, we found that SQ109 treatment activates p38 MAPK in lung-resident macrophages that are considered the primary niche for *M.tb* replication (Fig. 4b, d). Collectively, these data suggested that SQ109 treatment mediates inflammatory responses and apoptosis in macrophages via the MAPK and JNK signaling pathways.

## Discussion

In recent years, SQ109 has emerged as a promising drug against a variety of pathogens such as *M. tuberculosis, Helicobacter pylori*, *Clostridium difficile*, *Neisseria gonorrhoeae* etc.^16,54,55^. Apart from bacterial diseases, SQ109 has been proposed as a potent drug against Chagas disease caused by *Trypanosoma cruzi*^55^. It has been reported that SQ109 has antiproliferative and ultrastructural effects on three life cycle stages i.e., trypomastigotes, epimastigotes, and amastigotes of *T. cruzi*^55^. There are other reports which suggest that SQ109 inhibits proliferation of *Leishmania donovani* via disruption of the intracellular Ca^2+^ homeostasis along with collapsing the electrochemical potential of mitochondria^56^. In addition to these, it also induces rapid damage to the acidocalcisomes, an essential organelle involved in the bioenergetics of the parasite^56^. In case of TB infection, SQ109 reduces the likelihood of developing TB resistance as it has multiple targets. Additionally, studies have shown that antitubercular antibiotics such as Bedaquiline can interfere with an individual’s immune system, either indirectly through dysbiosis of their gut microbiota or directly by modulating the performance of their immune cells^24,25^. Therefore, in an attempt to decipher the effects of SQ109 treatment on immune responses of host during *tuberculosis* infection, we studied the dynamics of cytokines and effector immune cells in murine model of tuberculosis. For the first time, we reported the immune modulatory effect of SQ109 along with the mechanism through which it targets MAPK and JNK pathways, leading to M1 polarization and apoptosis. Previous studies have shown that SQ109 has a 99% inhibitory effect^6,16^ on *M.tb*, which is congruent with our results indicating that SQ109 treatment significantly reduces bacterial load in a murine model of TB by 1.5 logs. Here, we report that SQ109 drastically reduces bacterial burden in macrophages infected with MDR and XDR TB strains. Concomitantly, we also found that SQ109 has synergistic effects with isoniazid and reduces bacterial load in H37Rv-infected macrophages, which is in agreement with previous studies using conventional TB treatment protocols^12–14^.

The extent of host susceptibility to *M.tb* infection correlates with the dynamics of pro- and anti-inflammatory responses, which are critical for T helper cell differentiation^33,57^. It is well established that a defect in Th1 immune responses leads to increased susceptibility to *M.tb* infection^26^. Furthermore, we report that SQ109 treatment significantly increases the prevalence of IFN-γ and IL-17-producing CD4^+^ and CD8^+^ T cells. This finding suggests that SQ109 enhances protective immunity against both *M.tb* active infection and re-infection and has the potential to reverse the immune dampening effects of antibiotics used in TB treatment^4^.

Using RT-PCR and FACS techniques, we showed that SQ109 treatment in mice infected with H37Rv significantly upregulates the production of pro-inflammatory cytokines such as IL-6, IL-12, IFN-γ, and IL-1β, at both the mRNA and protein levels. Previous studies have shown that mice deficient in some of these cytokines succumb to *M.tb* infection^39–41^. Collectively, our study shows that SQ109 is an antibacterial agent with the potential to modulate host immune responses and enhance protection against *M.tb* infection.

Our findings suggest that SQ109 targets macrophages, which are known to play critical roles in inflammation. Interestingly, for the first time we report that the SQ109 treatment polarizes macrophages towards the M1 phenotype. M1-type macrophages are pro-inflammatory and provide protective immunity against *M.tb* infection^43,44,58^, whereas M2-type macrophages, which largely produce anti-inflammatory cytokines, promote *M.tb* replication^59^.

Tuberculosis is a disease of both infection and inflammation. A very recent study has shown the importance of M1 macrophages in *M.tb*. elimination from host^60^. It has been shown that during early stage of *M.tb*. infection, the differentiation of interstitial macrophages is towards M1 phenotype^60^. However, those M1 macrophages transformed to M2 macrophages during infection by early secretory antigenic target (ESAT-6), which is one of the major virulence factors of *M.tb*. and provides favorable environments for *M.tb*. survival. M2 macrophages have suppressive antibacterial response against *M.tb*. with elevated lipid catabolism. Thus, this transformation processes from M1 to M2 phenotype by *M.tb*. are budding immunological targets of tuberculosis.

Additionally, several studies reported that the SQ109 at 25 mg/kg of concentration does not impart any noticeable side effects like irritation at the site of injection, inability to move, ataxia, ruffled fur, tremors, emesis, convulsions, diarrhea, and acute death in murine model of tuberculosis^7^. Early phase trials of SQ109 on smear positive pulmonary TB patients have shown that SQ109 is a safe drug and well tolerated up to 300 mg/kg of dose^13^. Only mild to moderate dose dependent gastrointestinal complaints were noticed^13^. Hence, SQ109 or a similar drug that potentially polarizes macrophage towards M1 phenotype is highly desirable^60^. Our results demonstrated that SQ109 induces apoptosis in H37Rv-infected macrophages. These data are in agreement with a recent report by Lam et al., showing that predominance of M2-type macrophages inhibits apoptosis in *M.tb*-infected macrophages, thus enhancing *M.tb* survival in the host^45^. Lastly, our mechanistic studies revealed that SQ109 treatment activates the MAPK signaling pathway, which play pivotal role in the induction of pro-inflammatory cytokines^61,62^. MAPK activation in macrophages generates multiple effector molecules with anti-mycobacterial activity and also facilitates protective host immunity during *M.tb* infection^63,64^. We also found that SQ109 treatment activates the JNK pathway, which is associated with M1 polarization^49^ and apoptosis^65^.

**Fig. 5:**
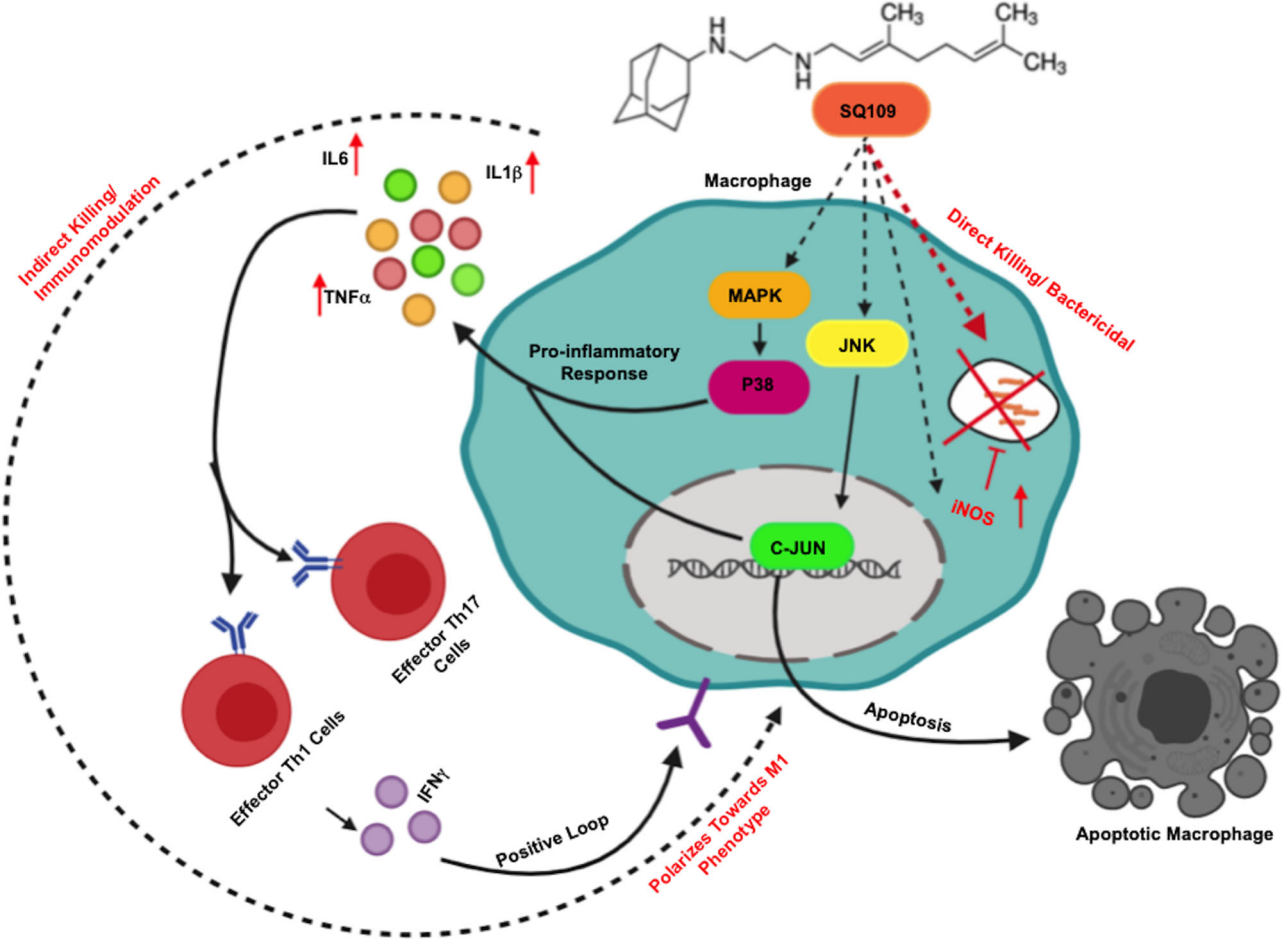
Proposed mechanism of action of SQ109. SQ109 has a dual mode of action, with direct bactericidal activity and immunomodulatory properties. SQ109 activates the MAPK and JNK pathways in macrophages, leading to the induction of pro-inflammatory M1-type macrophage responses and apoptosis. Pro-inflammatory responses further modulate the dynamics of T helper cells, which imparts further protection to the host. SQ109 induces production of iNOS, which further mediates the killing of intracellular mycobacteria.

Collectively, our findings revealed that SQ109 dramatically modulates host immunity and augments antibacterial immune responses against both drug-sensitive and resistant strains of *M.tb* (Fig. 5).

Therefore, a combination treatment of SQ109 with conventional anti-tubercular drugs could serve as an improved treatment modality for TB. However, the effect of SQ109 on dormant forms of *M.tb* that resides in mesenchymal stem cells and generally do not responds to antibiotics (latent infection of tuberculosis) remains to be established.

## Methods

### Ethical statement

In vivo experiments were executed as per the guidelines of the Institutional Animal Ethics Committee of ICGEB (New Delhi, India) and the DBT (Department of Biotechnology). All mice used for the experiments were ethically sacrificed by asphyxiation using carbon dioxide according to institutional and DBT protocols.

### Mice

C57BL/6 female mice of 6–8 weeks of age were obtained from ICGEB. These mice were maintained at the central animal facility of the ICGEB. All mice were group housed in IVC cages (cage dimension 425 ×x 266 × 155 mm) and total floor area were 820 cm^2^ with corncob bedding. Gamma irradiated, pelleted, non-contaminated, and nutritionally adequate food and autoclaved, fresh, potable drinking water were provided ad libitum. In-house temperature was maintained at 19–25 °C with relative humidity 30–70%, light intensity-130–325 Lux, noise less than 60 dB, photoperiod −12 h light and 12 h Dark phase and air changes 10–15 per hour. Enrichments materials were provided along with bedding material such as autoclaved intact tissue papers, small igloo etc. All mice were treated humanely as per Animal care protocols.

### Bacterial strains

The *M.tb* strain H37Rv (ATCC 25618) was provided by the Colorado State University repository. The multi drug-resistant (MDR-Jal2261; resistant to ethambutol, isoniazid and rifampicin) and extensively drug-resistant (XDR MYC 431) *M.tb* strains were a generous gift from Dr. KVS Rao (ICGEB). All mycobacterial strains were grown in 7H9 (Middlebrooks, Difco^TM^, USA) medium, which was supplemented with 0.05% Tween 80, 0.2% glycerol and 10% albumin, dextrose, and catalase enrichment medium (OADC; Difco, USA) The cultures were maintained up to mid-log phase and were then aliquoted in 20% glycerol and stored at −80 °C. These cryopreserved stocks were used for all experiments.

### RT PCR primers and reagents

Real-time quantitative RT-PCR analysis was performed using Bio-Rad Real-Time thermal cycler (Bio-Rad, USA). The reaction was set up according to the manufacturer’s protocol. The enzyme reverse transcriptase and SYBR green Master mix were procured from Bio-Rad. The relative expression level of mRNAs was normalized to that of internal control GAPDH by using 2-DDCt cycle threshold method. The primers used for the study (Supplementary Table 1) were purchased from Integrated DNA technologies (IDT), USA.

### Isolation of peritoneal macrophages from mice

Sterile thioglycolate (2 ml; Brewer modified, BBL, BD Biosciences: 4% w/v in water) was administered intraperitoneally into 6- to 8-week-old C57BL/6 mice^66^. Macrophages were isolated by peritoneal lavage 5 days after injection. Isolated macrophages were washed with 1X PBS and then re-suspended in 10% RPMI-1640 (Gibco, Invitrogen, UK) supplemented with fetal bovine serum^66^. Viable macrophages were counted using a hemocytometer by trypan blue dye exclusion method. 1 × 10^6^ cells were seeded in 12-well plates whereas 8 × 10^6^ cells were seeded in 100 mm-plates with 10% RPMI-1640. Cultured cells were maintained for 8 h for adherence. The non-adherent cells were washed away using 1X PBS.

### In vitro infection and CFU estimation

Peritoneal macrophages isolated from C57BL/6 mice were infected with exponentially growing *M.tb* H37Rv cultures at an MOI of 10 for 4 h^67^. For this purpose, mycobacterial stocks were revived from −80 °C freezer. Post revival, single cell suspension was prepared by passing it through a 26-gauge syringe ten times followed by passing through 30-gauge syringe. Cells were then incubated for an additional 2 h with RPMI media containing 100 μg/ml gentamycin to eliminate non-phagocytosed mycobacteria. Infected macrophages were then washed twice with PBS followed by culture in media containing 10 μg/ml gentamycin for the entire duration of the experiment^67^. For drug treatment, two different concentrations, 0.39 μg/ml and 0.78 μg/ml, of SQ109 were used for H37Rv and MDR/XDR strains of *M.tb*, respectively^16^. After the specified time points, cells were lysed using 0.06% SDS in PBS and three dilutions of the lysate were used for plating. 7H11 agar plates were used for plating and incubated at 37 °C for 21–28 days for counting the colony forming units (CFUs).

### *M.tb* aerosol infection of mice and quantification of bacterial burden by CFU estimation

Mice were infected in accordance with the low-dose aerosol infection model using Wisconsin Madison aerosol chamber (University of Wisconsin, Madison, USA). This instrument was connected with a pre-calibrated nebulizer to deliver a total of ~110 CFU to the lungs of each mouse. For this purpose, mycobacterial stocks were recovered from −80 °C freezer. Post revival, single cell suspension was prepared by passing it through a 26-gauge syringe ten times followed by passing through 30 –gauge syringe. Fifteen milliliters of the bacterial cell suspension (10 × 10^6^ cells per ml) was placed in the nebulizer attached to the Madison aerosol chamber. At 24 h post-aerosol challenge, three randomly selected mice were sacrificed for quantification of bacillus delivery to organs. Organs were homogenized in sterile PBS containing 0.05% Tween 80 and plated onto 7H11 Middlebrook plates containing 10% OADC. Three different dilutions (undiluted, 10-fold, and 100-fold) were plated on 7H11 plates and incubated at 37 °C for 21 days. CFUs were counted and pathogen burden in lung and spleen were estimated.

### Drug administration

10 mg/kg of SQ109 (Sigma, USA, Cat number SML1309) in 100 μl of PBS was injected intraperitoneally throughout the treatment phase, whereas the controls were provided with vehicle only.

### Flow cytometry: surface and intracellular staining

For cell surface staining, spleens were isolated from mice and macerated in ice cold RPMI 1640 (Gibco, Invitrogen, UK) with 10% FBS using frosted slides to form single cell suspension. Cells were incubated with RBC lysis buffer for 3–5 min at room temperature in order to lyse RBCs present in cells harvested from spleen and washed with 10% RPMI 1640. Cells were counted and 1 x 10^6^ cells per well were cultured in 12 well plates (NUNC, USA) followed by activation by

*M.tb* H37Rv Complete Soluble Antigen (CSA) overnight. Cells were washed twice with fluorescence-activated cell sorter (FACS) buffer and then stained with antibodies directed against surface markers and 7 AAD (Biolegend, USA). 7 AAD viability dye was used to discriminate live cell populations. Post addition of antibodies (Supplementary Table 2), cells were incubated at room temperature for 40 min for optimum staining. Post staining, cells were washed again using FACS buffer and resuspended in FACS buffer. For intracellular staining, 1 × 10^6^ cells per well were cultured in 12 well plates (NUNC, USA) and activated with *M.tb* H37Rv Complete Soluble Antigen (CSA) overnight with 5 μg/ml Brefeldin-A (Biolegend, USA) being added to the cells during the last 6 h of culture. Cells were washed twice with FACS buffer and post surface staining, cells were fixed with 100 µl of fixation buffer (Biolegend, USA) for 30 min. Cells were further washed twice with permeabilization buffer (Biolegend, USA) and subsequently suspended in 200 µl of permeabilization buffer followed by staining with fluorescently-conjugated antibodies. After staining, cells were washed again with FACS buffer and resuspended in FACS buffer. Compensation for all the lasers after running standard BD-CS&T beads were performed. Cells stained with single color/fluorochrome were used for compensation followed by FMO control to compensate overlapping and spilling of the fluorescence. The intensity of fluorescence was measured using flow cytometry (BD FACS Canto^TM^ II; BD) and results were analyzed using Flow Jo (Tree star, USA).

### Histology

Lung tissues were dissected out and fixed using 10% buffered formalin. Tissues were embedded in 5 µm-thick paraffin for sectioning. Sections were then stained with a nuclear dye (Hematoxylin) and counter-stained with Eosin. Ziehl–Neelsen stain (AFB) dye was used for bacillus staining. Slides were analyzed under a light microscope. To calculate the granuloma score, a minimum of 5 fields was screened for each mouse in each group.

### Western blotting

For the ex vivo experiments, peritoneal macrophages were treated with SQ109 (0.39 μg/ml) at different time points (30, 60, and 90 min). For the in vivo experiments, lung tissues were isolated from H37Rv-infected and SQ109-treated mice (10 mg/kg daily) at 60 days post-infection. Tissues were washed with PBS, homogenized, and centrifuged. For both ex-vivo and in-vivo experiments, the cell lysates were prepared using lysis buffer (500 mM NaCl, 1 mM EDTA, 20 mM Tris-HCl pH 8.0, 1 mM PMSF, 0.25% Triton X 100, 1 mM dithiothreitol, and 1X Phosphatase inhibitor cocktail [88266; Thermo fisher scientific]). 10% SDS-polyacrylamide gel was used for gel electrophoresis and proteins were transferred onto nitrocellulose membranes. Blocking was performed with 5% BSA in PBST (PBS with 0.1% Tween 20) for 1 h. Antibodies against p38 MAPK, phospho-p38 MAPK, p42/44 ERK, phospho-p42/44 ERK, c-JUN, and phospho-c-JUN were used at a dilution of 1:1000 as recommended by the manufacturer (Cell Signaling Technologies, USA). For secondary antibody, goat-raised anti-rabbit immunoglobulin G-conjugated horseradish peroxidase (Cell Signaling Technologies, USA) was used at a dilution 1:2000. GAPDH was used as loading control.

### Macrophage polarization assay

8 × 10^6^ peritoneal macrophages were seeded in 100-mm plates and incubated at 37 °C overnight. Cells were washed with 1x PBS followed by addition of RPMI media supplemented with 10% fetal bovine serum. Cells were then treated with or without 0.39 µg/ml of SQ109. After 24 h of treatment, cells were washed with PBS and harvested to study the polarization markers using RT-PCR.

### Statistics and reproducibility

All results were obtained from at least three independent experiments. Statistical significance was tested using multiple/unpaired *t*-test unless specified otherwise. *p* < 0.05 was accepted as an indication of statistical significance. For Fig. 1, *n* = 5 biological replicates were used for two independent experiments. Statistical significance was determined using unpaired *t*-test. For Fig. 2, statistical significance was determined using multiple *t*-test. *n* = 5 biological replicates were used for two independent experiments. For Fig. 3, *n* = 3 biological replicates were used for three independent experiments. Statistical significance was tested using multiple/unpaired *t*-test unless specified otherwise. For Fig. 5, *n* = 3 biological replicates were used for three independent experiments except for Fig. 4b, where only two independent experiments were undertaken. Statistical significance was tested using unpaired *t*-test.

### Reporting summary

Further information on research design is available in the Nature Research Reporting Summary linked to this article.

## Supplementary information

Supplementary Information

Description of Additional Supplementary Files

Supplementary Data 1

Reporting Summary

## Data Availability

All data generated or analyzed during this study are included in this published article (and its supplementary information files like gating strategies, supporting RT-PCR data and unedited gel picture of the western blot images). Complete source data file has been provided as Supplementary Data 1.
