# Supplementary material for: The 1, 2-ethylenediamine SQ109 protects against tuberculosis by promoting M1 macrophage polarization through the p38 MAPK pathway
Source: Commun Biol. 2022 Jul 28;5:759. doi: 10.1038/s42003-022-03693-2 (PMC9334294; doi:10.1038/s42003-022-03693-2)
Supplement: Supplementary file 1 — Supplementary Information [file 42003_2022_3693_MOESM1_ESM.pdf]

Supplementary Information

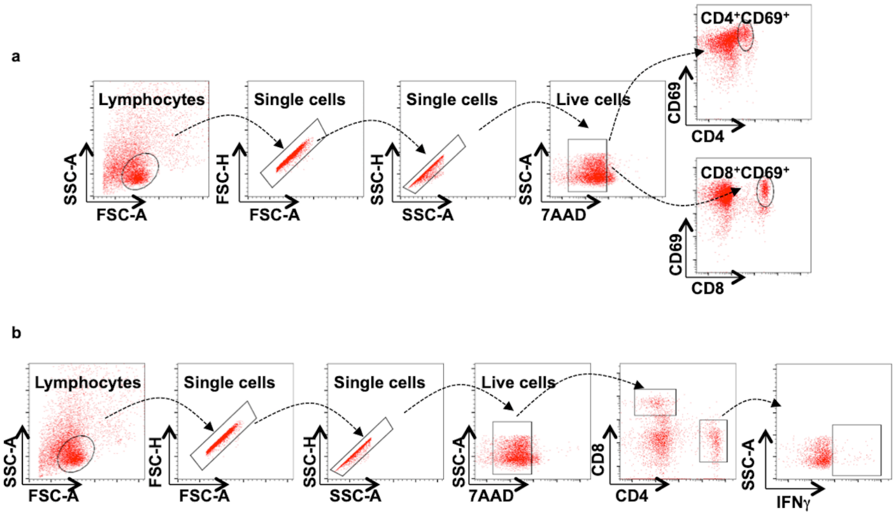

Supplementary Figure 1: **(a)** Gating strategy employed to quantify the T cell activation. **(b)** Gating strategy employed to quantify the level of intracellular cytokines.

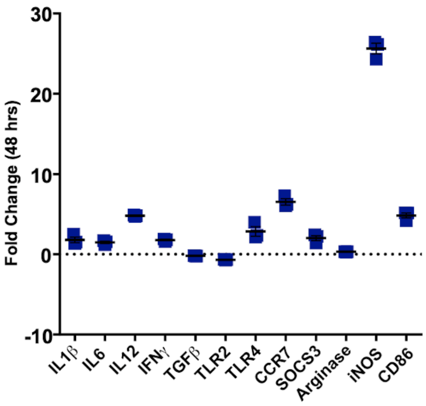

Supplementary Figure 2: RT-PCR data showing fold-changes in the expression of cytokines and macrophage polarizing markers in SQ109-treated macrophages compared to control macrophages post 48 hrs of treatment.

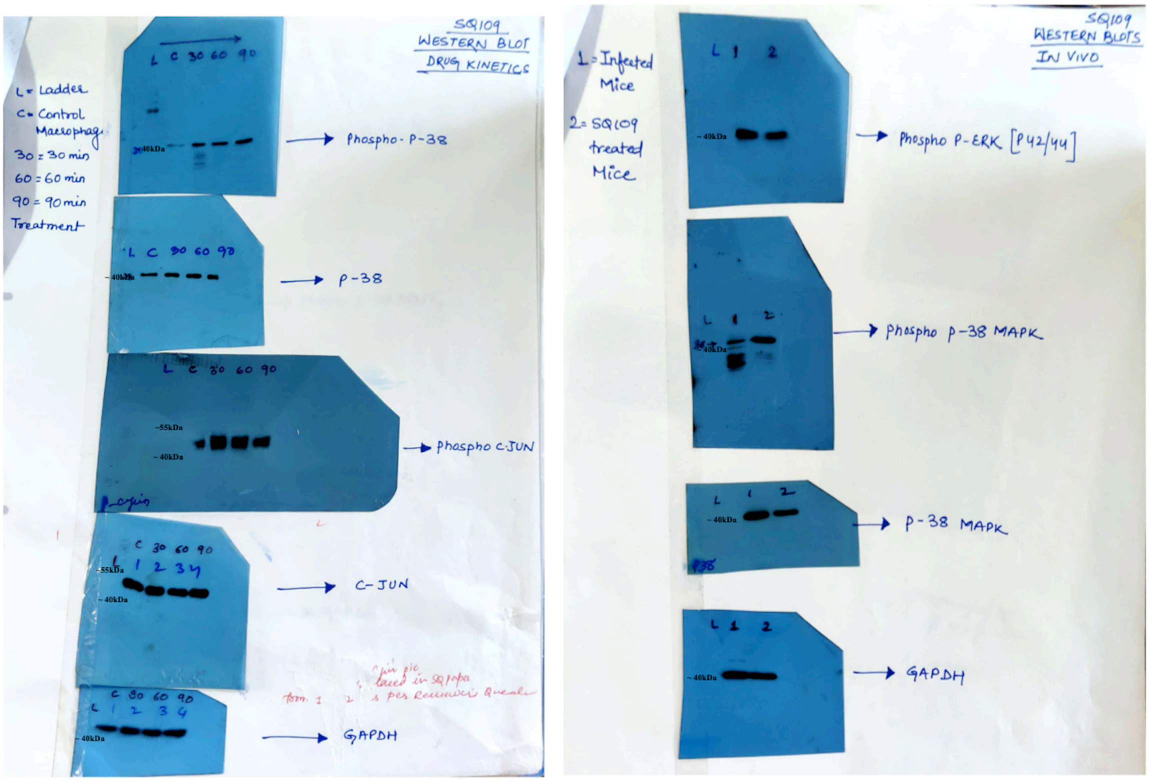

Supplementary Figure 3: Raw uncropped gel images of the western blots.

Supplementary Table 1: List of Primers Used for the Study

S.No.	Gene	Forward	Reverse	References
1.	iNOS	CGAAACGCTTCACTTCCAA	TGAGCCTATATTGCTGTGGCT	[66]
2.	Arginase1	AACACGGCAGTGGCTTTAACC	GGTTTTCATGTGGCGCATTC	[67,68]
3.	CD86	TGTTTCCGTGGAGACGCAAG	TTGAGCCTTTGTAAATGGGCA	[69]
4.	IL-1β	CACCTTCTTTTCCTTCATCTTTG	GTCGTTGCTTGTCTCTCCTTGTA	[70]
5.	IL-6	TGATGGATG CTTCCAAACTG	GAGCATTGG AAGTTGGGGTA	[71]
6.	TNFα	ACTGAACTT CGGGGTGATTG	GCTTGGTGG TTTGCTACGAC	[71]
7.	TLR2	TCTGGGCAGTCTTGAACATTT	AGAGTCAGGTGATGGATGTCG	[72]
8.	TLR4	CAAGGGATAAGAACGCTAGA	GCAATGTCTCTGGCAGGTGTA	[73]
9.	SOCS3	CGAAGCACG CAGCCAGTT	TCCGTGGGT GGCAAAGAA	[74]
10.	IFNγ	TGAACGCTACACACTGCATCTTGG	CGACTCCTTTTCCGCTTCCTGAG	[75]
11.	TGFβ	GAGGTCACCCGCGTGCTA	TGTGTGAGATGTCTTTGGTTTTCTC	[76]
12.	IL-10	CAGCCGGGAAGACAATAACTG	CCGCAGCTCTAGGAGCATGT	[76]
13.	IL-12	AAGCTCTGCATCCTGCTTCAC	GATAGCCCATCACCCTGTTGA	[76]
14.	GAPDH	AAGGGCTCATGACCACAGTC	CAGGGATGATGTTCTGGGCA	[77]

Supplementary Table 2: List of Antibodies Used for the Study

S.No.	Antibodies	Clone	Catalogue No.	Company
1.	Anti-CD8<br>(–FITC, -APC -H7, - PerCP-Cy5.5 or – APC)	53 - 6.7	553030, 560247,<br>551162, 553035	BD Biosciences, USA
2.	CD44<br>(-APC)	IM7	559250	BD Biosciences, USA
3.	CD4<br>(-FITC, -PerCP-Cy5.5 or –APC)	GK1.5,<br>RM4 -5	553729, 553046,<br>553051, 561115	BD Biosciences, USA
4.	IFN-γ<br>(-APC)	XMG1.<br>2	554413	BD Biosciences, USA
5.	IL-6<br>(-PE)	MPS -<br>20F3	554401	BD Biosciences, USA
6.	IL-4<br>(-PE)	11B11	554435	BD Biosciences, USA
7.	IL-12<br>(-PE)	C15.6	554479	BD Biosciences, USA
8.	IL-10<br>(–APC)	JES5-<br>16E3	554468	BD Biosciences, USA
9.	TNF-α<br>(-PE)	MP6-<br>XT22	554419	BD Biosciences, USA
10.	IL-17<br>(–PE)	O79-289	561627	BD Biosciences, USA
11.	CD69<br>(-PE)	H1.2F3	553237	BD Biosciences, USA
12.	TGF –β<br>(-APC)	TW7-<br>16B4	141406	Biolegend, USA

66. Wang, C., Dong, C. & Xiong, S. IL-33 enhances macrophage M2 polarization and protects mice from CVB3-induced viral myocarditis. *J Mol Cell Cardiol* **103**, 22-30 (2017).
67. Xu, H., *et al.* Uncoupling lipid metabolism from inflammation through fatty acid binding protein-dependent expression of UCP2. *Mol Cell Biol* **35**, 1055-1065 (2015).
68. Yeramian, A., *et al.* Arginine transport via cationic amino acid transporter 2 plays a critical regulatory role in classical or alternative activation of macrophages. *J Immunol* **176**, 5918-5924 (2006).
69. Morris, D.L., *et al.* Adipose tissue macrophages function as antigen-presenting cells and regulate adipose tissue CD4+ T cells in mice. *Diabetes* **62**, 2762-2772 (2013).
70. Heijnen, B.F., Van Essen, H., Schalkwijk, C.G., Janssen, B.J. & Struijker-Boudier, H.A. Renal inflammatory markers during the onset of hypertension in spontaneously hypertensive rats. *Hypertens Res* **37**, 100-109 (2014).
71. Jiang, W., Wang, Y., Sun, W. & Zhang, M. Morin Suppresses Astrocyte Activation and Regulates Cytokine Release in Bone Cancer Pain Rat Models. *Phytother Res* **31**, 1298-1304 (2017).
72. Brown, H.J., Lock, H.R., Sacks, S.H. & Robson, M.G. TLR2 stimulation of intrinsic renal cells in the induction of immune-mediated glomerulonephritis. *J Immunol* **177**, 1925-1931 (2006).
73. Wolfs, T.G., *et al.* In vivo expression of Toll-like receptor 2 and 4 by renal epithelial cells: IFN-gamma and TNF-alpha mediated up-regulation during inflammation. *J Immunol* **168**, 1286-1293 (2002).
74. Lu, S., Li, D., Xi, L. & Calderone, R. Interplay of interferon-gamma and macrophage polarization during Talaromyces marneffei infection. *Microb Pathog* **134**, 103594 (2019).
75. He, S., *et al.* Immune-related GTPase M (IRGM1) regulates neuronal autophagy in a mouse model of stroke. *Autophagy* **8**, 1621-1627 (2012).
76. Lin, J., *et al.* Cyr61 induces IL-6 production by fibroblast-like synoviocytes promoting Th17 differentiation in rheumatoid arthritis. *J Immunol* **188**, 5776-5784 (2012).
77. Kalagara, R., *et al.* Identification of stable reference genes for lipopolysaccharide-stimulated macrophage gene expression studies. *Biol Methods Protoc* **1**, bpw005 (2016).
