# Supplementary material for: The 1, 2-ethylenediamine SQ109 protects against tuberculosis by promoting M1 macrophage polarization through the p38 MAPK pathway
Source: Commun Biol. 2022 Jul 28;5:759. doi: 10.1038/s42003-022-03693-2 (PMC9334294; doi:10.1038/s42003-022-03693-2)
Supplement: Supplementary file 2 — Description of Additional Supplementary Files [file 42003_2022_3693_MOESM2_ESM.pdf]

Description of Additional Supplementary Files

File name: Supplementary Data 1
Description: The source data behind the figures and graphs in the paper.
