# Supplementary material for: The 1, 2-ethylenediamine SQ109 protects against tuberculosis by promoting M1 macrophage polarization through the p38 MAPK pathway
Source: Commun Biol. 2022 Jul 28;5:759. doi: 10.1038/s42003-022-03693-2 (PMC9334294; doi:10.1038/s42003-022-03693-2)
Supplement: Supplementary file 4 — Reporting Summary [file 42003_2022_3693_MOESM4_ESM.pdf]

nature portfolio

Corresponding author(s): Gobardhan Das
Ved Prakash Dwivedi

Last updated by author(s): Jun 2, 2022

Reporting Summary

Nature Portfolio wishes to improve the reproducibility of the work that we publish. This form provides structure for consistency and transparency in reporting. For further information on Nature Portfolio policies, see our Editorial Policies and the Editorial Policy Checklist.

Statistics

For all statistical analyses, confirm that the following items are present in the figure legend, table legend, main text, or Methods section.

n/a	Confirmed	
☐	☒	The exact sample size (*n*) for each experimental group/condition, given as a discrete number and unit of measurement
☐	☒	A statement on whether measurements were taken from distinct samples or whether the same sample was measured repeatedly
☐	☒	The statistical test(s) used AND whether they are one- or two-sided<br>*Only common tests should be described solely by name; describe more complex techniques in the Methods section.*
☐	☒	A description of all covariates tested
☐	☒	A description of any assumptions or corrections, such as tests of normality and adjustment for multiple comparisons
☐	☒	A full description of the statistical parameters including central tendency (e.g. means) or other basic estimates (e.g. regression coefficient) AND variation (e.g. standard deviation) or associated estimates of uncertainty (e.g. confidence intervals)
☐	☒	For null hypothesis testing, the test statistic (e.g. *F*, *t*, *r*) with confidence intervals, effect sizes, degrees of freedom and *P* value noted<br>*Give P values as exact values whenever suitable.*
☒	☐	For Bayesian analysis, information on the choice of priors and Markov chain Monte Carlo settings
☒	☐	For hierarchical and complex designs, identification of the appropriate level for tests and full reporting of outcomes
☒	☐	Estimates of effect sizes (e.g. Cohen's *d*, Pearson's *r*), indicating how they were calculated

Our web collection on statistics for biologists contains articles on many of the points above.

Software and code

Policy information about availability of computer code

Data collection: NA

Data analysis: NA

For manuscripts utilizing custom algorithms or software that are central to the research but not yet described in published literature, software must be made available to editors and reviewers. We strongly encourage code deposition in a community repository (e.g. GitHub). See the Nature Portfolio guidelines for submitting code & software for further information.

Data

Policy information about availability of data

All manuscripts must include a data availability statement. This statement should provide the following information, where applicable:

- Accession codes, unique identifiers, or web links for publicly available datasets
- A description of any restrictions on data availability
- For clinical datasets or third party data, please ensure that the statement adheres to our policy

NA

Human research participants

Policy information about studies involving human research participants and Sex and Gender in Research.

Reporting on sex and gender	NA
Population characteristics	NA
Recruitment	NA
Ethics oversight	NA

Note that full information on the approval of the study protocol must also be provided in the manuscript.

Field-specific reporting

Please select the one below that is the best fit for your research. If you are not sure, read the appropriate sections before making your selection.

☒ Life sciences ☐ Behavioural & social sciences ☐ Ecological, evolutionary & environmental sciences

For a reference copy of the document with all sections, see nature.com/documents/nr-reporting-summary-flat.pdf

Life sciences study design

All studies must disclose on these points even when the disclosure is negative.

Sample size	C57BL/6 mice (n =5) in each group were used in this study as per our previous work (Dwivedi et al., 2017; PMID: 28507951).
Data exclusions	supplementary figure 3 &4 excluded because those were related to previous reviewer comments(jbc) which was not needed here.
Replication	Three independent experiments were performed. However in case of in vivo experiments two independent experiments were performed.
Randomization	C57 BL/6 female mice were used for the experiments to avoid the genetic heterogeneity. As the aim of this study was not to see the impact of drug on male and female mice.
Blinding	NA

Reporting for specific materials, systems and methods

We require information from authors about some types of materials, experimental systems and methods used in many studies. Here, indicate whether each material, system or method listed is relevant to your study. If you are not sure if a list item applies to your research, read the appropriate section before selecting a response.

Materials & experimental systems

n/a	Involved in the study	
☐	☒	Antibodies
☒	☐	Eukaryotic cell lines
☒	☐	Palaeontology and archaeology
☐	☒	Animals and other organisms
☒	☐	Clinical data
☒	☐	Dual use research of concern

Methods

n/a	Involved in the study	
☒	☐	ChIP-seq
☐	☒	Flow cytometry
☒	☐	MRI-based neuroimaging

Antibodies

Antibodies used

1. Anti-CD8
(–FITC, -APC -H7, - PerCP-Cy5.5 or – APC)
53 - 6.7 553030, 560247, 551162, 553035 BD Biosciences, USA
2. CD44
(-APC)
IM7 559250 BD Biosciences, USA

3. CD4
(-FITC, -PerCP-Cy5.5 or –APC)
GK1.5, RM4 -5 553729, 553046, 553051, 561115 BD Biosciences, USA
4. IFN-γ
(-APC)
XMG1.2 554413 BD Biosciences, USA
5. IL-6
(-PE)
MPS -20F3 554401 BD Biosciences, USA
6. IL-4
(-PE)
11B11 554435 BD Biosciences, USA
7. IL-12
(-PE)
C15.6 554479 BD Biosciences, USA
8. IL-10
(–APC)
JES5-16E3 554468 BD Biosciences, USA
9. TNF-α
(-PE)
MP6-XT22 554419 BD Biosciences, USA
10. IL-17
(–PE)
O79-289 561627 BD Biosciences, USA
11. CD69
(-PE)
H1.2F3 553237 BD Biosciences, USA
12. TGF –β
(-APC)
TW7-16B4 141406
Biolegend, USA

Validation: The antibodies were validated in previous studies (Dwivedi et al., 2017; PMID: 28507951, Singh et al., 2021 PMID: 34415976).

Animals and other research organisms

Policy information about studies involving animals; ARRIVE guidelines recommended for reporting animal research, and Sex and Gender in Research

Laboratory animals: C57BL/6 female mice of 6–8 weeks of age

Wild animals: NA

Reporting on sex: NA

Field-collected samples: NA

Ethics oversight: In vivo experiments were executed as per the guidelines of the Institutional Animal Ethics Committee of ICGEB (New Delhi, India) and the DBT (Department of Biotechnology).

Note that full information on the approval of the study protocol must also be provided in the manuscript.

Flow Cytometry

Plots

Confirm that:

☒ The axis labels state the marker and fluorochrome used (e.g. CD4-FITC).

☒ The axis scales are clearly visible. Include numbers along axes only for bottom left plot of group (a 'group' is an analysis of identical markers).

☒ All plots are contour plots with outliers or pseudocolor plots.

☒ A numerical value for number of cells or percentage (with statistics) is provided.

Methodology

Sample preparation: Detailed methodology of the sample preparation and data acquisition is mentioned in the method section.

Instrument: BD FACS LSR Fortessa X20

Software	For acquisition: BD FACS Diva For analysis: Flowjo 10.6.1 (tree star)
Cell population abundance	No sorting was performed
Gating strategy	Detailed gating strategy is provided as supplementary figure 1

☒ Tick this box to confirm that a figure exemplifying the gating strategy is provided in the Supplementary Information.
